# Time-dependent effects of ipragliflozin on behaviour and energy homeostasis in normal and type 2 diabetic rats: continuous glucose telemetry analysis

**DOI:** 10.1038/s41598-017-12106-y

**Published:** 2017-09-19

**Authors:** Hiroyuki Iuchi, Masaya Sakamoto, Daisuke Matsutani, Hirofumi Suzuki, Yosuke Kayama, Norihiko Takeda, Susumu Minamisawa, Kazunori Utsunomiya

**Affiliations:** 1 Division of Diabetes, Metabolism and Endocrinology, Department of Internal Medicine, The Jikei University School of Medicine, Tokyo, Japan; 2 Department of Cardiology, The Jikei University School of Medicine, Tokyo, Japan; 3 Department of Cardiovascular Medicine, Graduate School of Medicine, The University of Tokyo, Tokyo, Japan; 4 Department of Cell Physiology, The Jikei University School of Medicine, Tokyo, Japan

## Abstract

Sodium–glucose cotransporter 2 (SGLT2) inhibitors are oral antidiabetic drugs that promote urinary glucose excretion. Conversely, they cause behavioural changes, such as hyperphagia, that result in a positive energy balance. The relationship between energy homeostasis and SGLT2 inhibitors-induced behavioural changes remains unclear. Here we show that ipragliflozin, a SGLT2 inhibitor, time-dependently affects behaviour and enhances energy expenditure in normal and type 2 diabetic Goto–Kakizaki (GK) rats, using continuous glucose telemetry. Alongside increased urinary glucose excretion, ipragliflozin increased total food and water intakes in normal and GK rats. In normal rats, ipragliflozin treatment acutely disturbed the circadian rhythms of food and water intakes, activity, and body temperature. Subsequently, these rhythms gradually returned towards a normal state. However, activity and body temperature remained suppressed. In GK rats, ipragliflozin did not affect circadian rhythms. Blood glucose values assessed by glucose telemetry were significantly reduced in both ipragliflozin-treated groups. Despite these behavioural and glycaemic changes, ipragliflozin significantly increased oxygen consumption during dark and light periods in both groups. Ipragliflozin reduced body weight in normal rats only. Thus, ipragliflozin decreases blood glucose beyond compensatory hyperphagia in normal and GK rats, resulting in enhanced basal energy expenditure, despite acutely altering circadian rhythms in normoglycaemic individuals.

## Introduction

Type 2 diabetes is becoming an increasingly common disorder. This change is connected with the increasing prevalence of obesity, which can drive type 2 diabetes^1,2^. Lifestyle interventions such as the control of body weight and food intake are essential to delay disease progression and improve glycaemic control in type 2 diabetes^3^. Furthermore, despite a number of anti-hyperglycaemic agents having recently become available, fewer than 50% of type 2 diabetes patients achieve the glycaemic goals recommended by the American Diabetes Association and European Association for the Study of Diabetes^4^.

Sodium–glucose cotransporter 2 (SGLT2) inhibitors are a newly developed class of antidiabetic drugs that promote the urinary excretion of glucose by inhibiting the reabsorption of glucose in renal proximal tubules^5^. SGLT2 inhibitors not only lower blood glucose but also reduce body weight in diabetic patients^6,7^. By contrast, SGLT2 inhibitors lead to compensatory hyperphagia in humans, seemingly due to calorie loss^8^. Consuming extra calories would be considered to attenuate the glucose-lowering effect of SGLT2 inhibitors and cause weight gain. In fact, in diet-induced obese rats, SGLT2 inhibitors-induced hyperphagia attenuates the reduction of body weight^9^. Moreover, since a decreased calorie intake leads to a lower basal energy expenditure^10^, the calorie loss induced by urinary glucose excretion may cause a lower energy expenditure. Food intake and urinary glucose excretion are important contributors to glucose and energy homeostasis, and changes in these parameters would cause changes in body weight. However, the relationship between energy homeostasis and SGLT2 inhibitors-induced behavioural changes remains unclear.

Considering the technical methods for measuring the response of blood glucose to SLGT2 inhibitors, while the technique of continuous glucose monitoring is easy to use in humans, continuous glucose measurement in rodent models has been technically difficult. Since any stress such as restraint or anaesthesia tends to increase blood glucose in rodent models^11^, a method for measuring the long-term continuous glycaemic profile under unrestrained and conscious conditions was needed.

Here we show that ipragliflozin, a SGLT inhibitor, time-dependently affects behaviour and enhances energy expenditure in normal Wistar and type 2 diabetic Goto–Kakizaki (GK) rats using continuous glucose telemetry, which is a newly-established technology for continuous glucose monitoring^12^.

## Results

### Ipragliflozin increased food and water intakes in normal and GK rats, and reduced activity and body temperature in normal rats, demonstrating altered circadian rhythms in the acute phase of treatment

**Figure 1:**
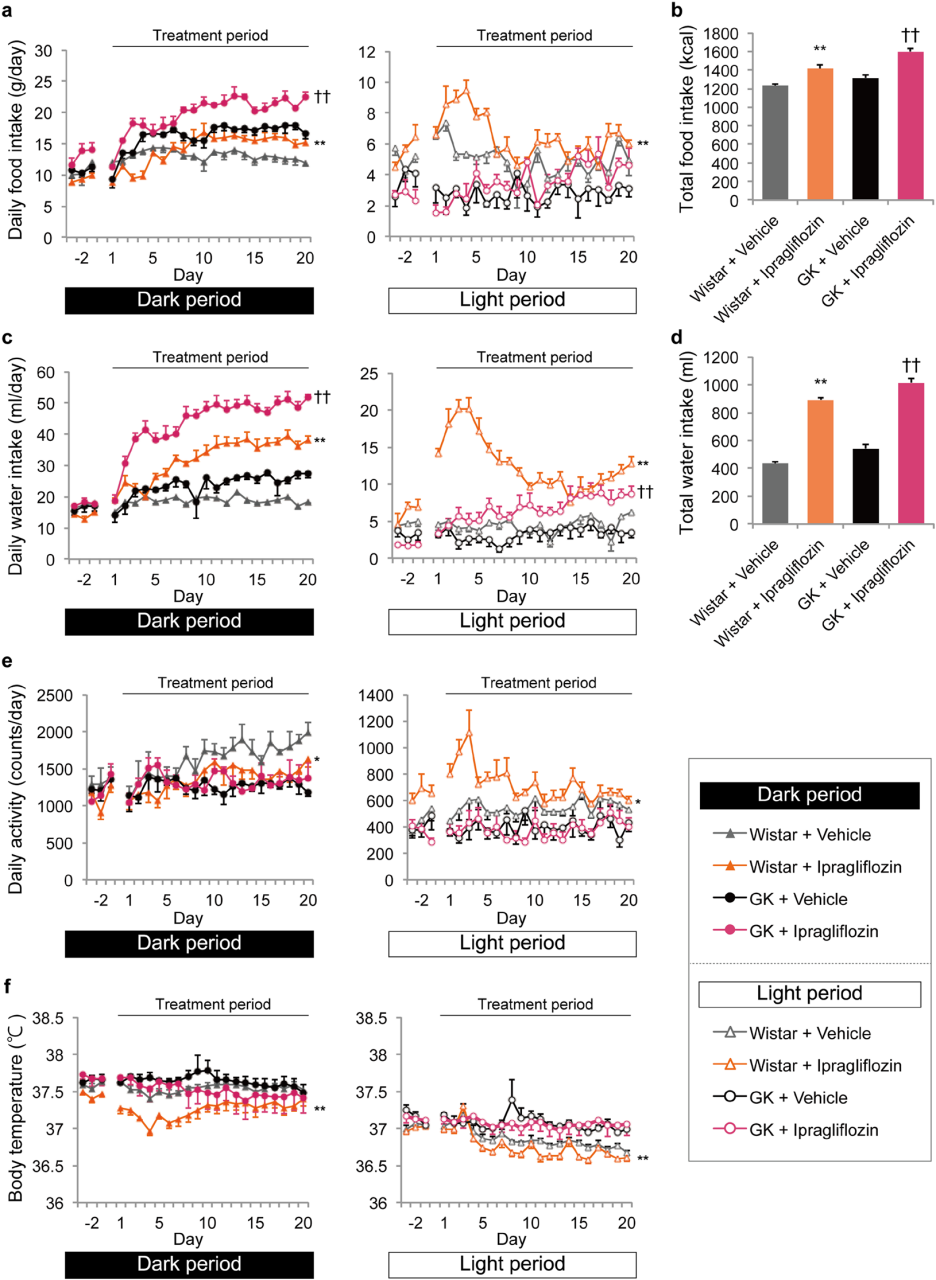
Effects of ipragliflozin on food and water intakes, activity, and body temperature during dark and light periods in normal and GK rats. (**a**) Daily food intake during dark (left) and light (right) periods. **P < 0.05 *vs*. Wistar + vehicle; dark period, days 1, 3, 4, 13, 15, 17, 18, and 20; light period, days −2, 3–6, 13, 16, and 18. ^††^ *P* < 0.05 *vs*. GK + vehicle; dark period, days −2, 3, 8–15, and 17–20. (**b**) Total food intake during the entire period, days 1–20. **P < 0.01 *vs*. Wistar + vehicle, ^††^ *P* < 0.01 *vs*. GK + vehicle. (**c**) Daily water intake during dark (left) and light (right) periods. **P < 0.05 *vs*. Wistar + vehicle; dark period, days −2, −1, 1, 3, and 5–20; light period, days −2, 1–13, and 16–20. ^††^ *P* < 0.05 *vs*. GK + vehicle; dark period, days 2–20; light period, days 6–8, 15, 17, 19, and 20. (**d**) Total water intake during the entire period, days 1–20. **P < 0.01 *vs*. Wistar + vehicle, ^††^ *P* < 0.01 *vs*. GK + vehicle. (**e**) Daily activity during dark (left) and light (right) periods. *P < 0.05 *vs*. Wistar + vehicle; dark period, days 16, 17, and 19; light period, days −3, 1, 2, 5, and 8. (**f**) Body temperature during dark (left) and light (right) periods. **P < 0.05 *vs*. Wistar + vehicle; dark period, days −1, 1–11, 13, 17, and 19; light period, days 8, 9, 11, 13, 15, 16, and 19. Values are expressed as mean ± standard error of the mean (s.e.m.). *n* = 4 per group.

Daily food intake during the dark period significantly increased through the latter half of the treatment period in both normal and GK rats (days 1, 3, 4, 13, 15, 17, 18, and 20, *P* < 0.05; days −2, 3, 8–15, and 17–20, *P* < 0.05). Daily food intake during the light period increased from the first half of the treatment period only in normal rats (days −2, 3–6, 13, 16, and 18; *P* < 0.05) (Fig. 1a). Considering the Wistar and GK rats separately, the circadian rhythm of food intake in the Wistar rats shifted from the dark to light periods in the acute phase of treatment (Supplementary Figure S1a). Total food intake during the entire treatment period was greater in the ipragliflozin-treated groups than in the vehicle-treated groups (Fig. 1b). The implantation of the telemetry device mainly affected the reduction of food intake in the pre-treatment period, compared with sham-operated GK rats (dark period, days −2, −1, 3, and 12, *P* < 0.05; light period, day −2, *P* < 0.05) (Supplementary Figure S2a).

Daily water intake during both the dark and light periods was significantly higher in the ipragliflozin-treated groups than in the vehicle-treated groups (Wistar during dark period, days −2, −1, 1, 3, and 5–20, *P* < 0.05; Wistar during light period, days −2, 1–13, and 16–20, *P* < 0.05; GK during dark period, days 2–20, *P* < 0.05; GK during light period, days 6–8, 15, 17, 19, and 20, *P* < 0.05) (Fig. 1c). In addition, in the ipragliflozin-treated normal rats, daily water intake during the light period in the acute phase of treatment first greatly increased and then later decreased in the latter half of the treatment period (Supplementary Figure S1b). Additionally, total water intake during the entire treatment period was greater in the ipragliflozin-treated groups than in the vehicle-treated groups (Fig. 1d).

Daily activity in the ipragliflozin-treated Wistar rats shifted from the dark to light periods in the acute phase of treatment (Supplementary Figure S1c). Moreover, daily activity during the dark period in the ipragliflozin-treated Wistar rats significantly decreased after the circadian rhythm normalised, compared with the vehicle-treated Wistar rats (dark period, days 16, 17, and 19, *P* < 0.05; light period, days −3, 1, 2, 5 and 8, *P* < 0.05). However, there was no difference in daily activity between treatment groups in GK rats (Fig. 1e).

Body temperature in the ipragliflozin-treated Wistar rats greatly decreased in the acute phase of treatment and decreased during both the dark and light periods in the latter half of the treatment period when compared with that of the vehicle-treated Wistar rats (dark period, days −1, 1–11, 13, 17, and 19, *P* < 0.05; light period, days 8, 9, 11, 13, 15, 16, and 19, *P* < 0.05) (Fig. 1f). In contrast, body temperature, including its circadian rhythm, did not change in GK rats.

### Ipragliflozin increased urine volume, urine glucose concentration, and ketone bodies in normal and GK rats

The clinical chemistry results are shown in Table 1. Glycated albumin and triglyceride were significantly reduced in the ipragliflozin-treated GK rats, compared with their levels in the vehicle-treated GK rats. In contrast, glycated albumin and triglyceride in Wistar rats did not significantly change with ipragliflozin treatment. Total ketone bodies, acetoacetone, and 3-hydroxybutyrate were significantly higher in the ipragliflozin-treated groups than in the vehicle-treated groups. Urine volume and urine glucose concentration were significantly higher in the ipragliflozin-treated groups than in the vehicle-treated groups.

**Table 1:** Metabolic and urinary variables in normal and GK rats treated with or without ipragliflozin.

	Wistar + Vehicle	Wistar + Ipragliflozin	GK + Vehicle	GK + Ipragliflozin
GA (%)	1.50 ± 0.05	1.53 ± 0.07	2.3 ± 0.07	1.9 ± 0.09^†^
HbA1c (%)	5.32 ± 0.13	5.31 ± 0.17	5.21 ± 0.12	5.465 ± 0.03
NEFA (μEq/L)	353.8 ± 25.5	464.3 ± 48.1	444.3 ± 18.3	761.3 ± 150.7
Triglyceride (mg/dL)	119.5 ± 12.4	99.2 ± 16.0	52.3 ± 7.4	31.0 ± 3.9^†^
Total cholesterol (mg/dL)	67.0 ± 3.5	58.2 ± 3.0	77.8 ± 5.2	78.8 ± 3.8
HDL cholesterol (mg/dL)	4.2 ± 0.3	5.2 ± 0.7	9.0 ± 0.9	7.0 ± 0.7
LDL cholesterol (mg/dL)	26.8 ± 1.4	25.3 ± 0.6	24.3 ± 1.8	29.0 ± 2.1
BUN (mg/dL)	19.8 ± 0.6	22.5 ± 0.6*	15.7 ± 1.2	16.1 ± 1.0
CRE (mg/dL)	0.33 ± 0.01	0.33 ± 0.01	0.26 ± 0.02	0.24 ± 0.00
Total ketone bodies (μmol/L)	315.7 ± 12.7	579.8 ± 70.0*	414.8 ±130.4	1483.3 ± 376.2^†^
Acetoacetone (μmol/L)	50.3 ± 2.6	118.8 ± 19.0*	91.5 ± 39.9	321.5 ± 74.8^†^
3-hydroxybutyrate (μmol/L)	265.3 ± 11.7	461.0 ± 52.9*	323.3 ± 92.7	1161.8 ± 301.9^†^
Urine volume (ml/day)	11.2 ± 0.5	26.9 ± 2.2**	9.6 ± 0.7	29.3 ± 3.6^††^
Urine glucose concentration (mg/dL)	31.8 ± 3.4	11260.0 ± 148.2**	588.3 ± 349.4	9401.0 ± 1647.7^††^

GA, glycated albumin; BUN, blood urea nitrogen; NEFA, nonesterified free fatty acids. Values are expressed as mean ± s.e.m. *P < 0.05, **P < 0.01 for Wistar + ipragliflozin vs. Wistar + vehicle, ^†^ *P* < 0.05, ^††^ *P* < 0.01 for GK + ipragliflozin vs. GK + vehicle. *n* = 4–6.

### Ipragliflozin decreased blood glucose values as assessed by glucose telemetry in normal and GK rats

**Figure 2:**
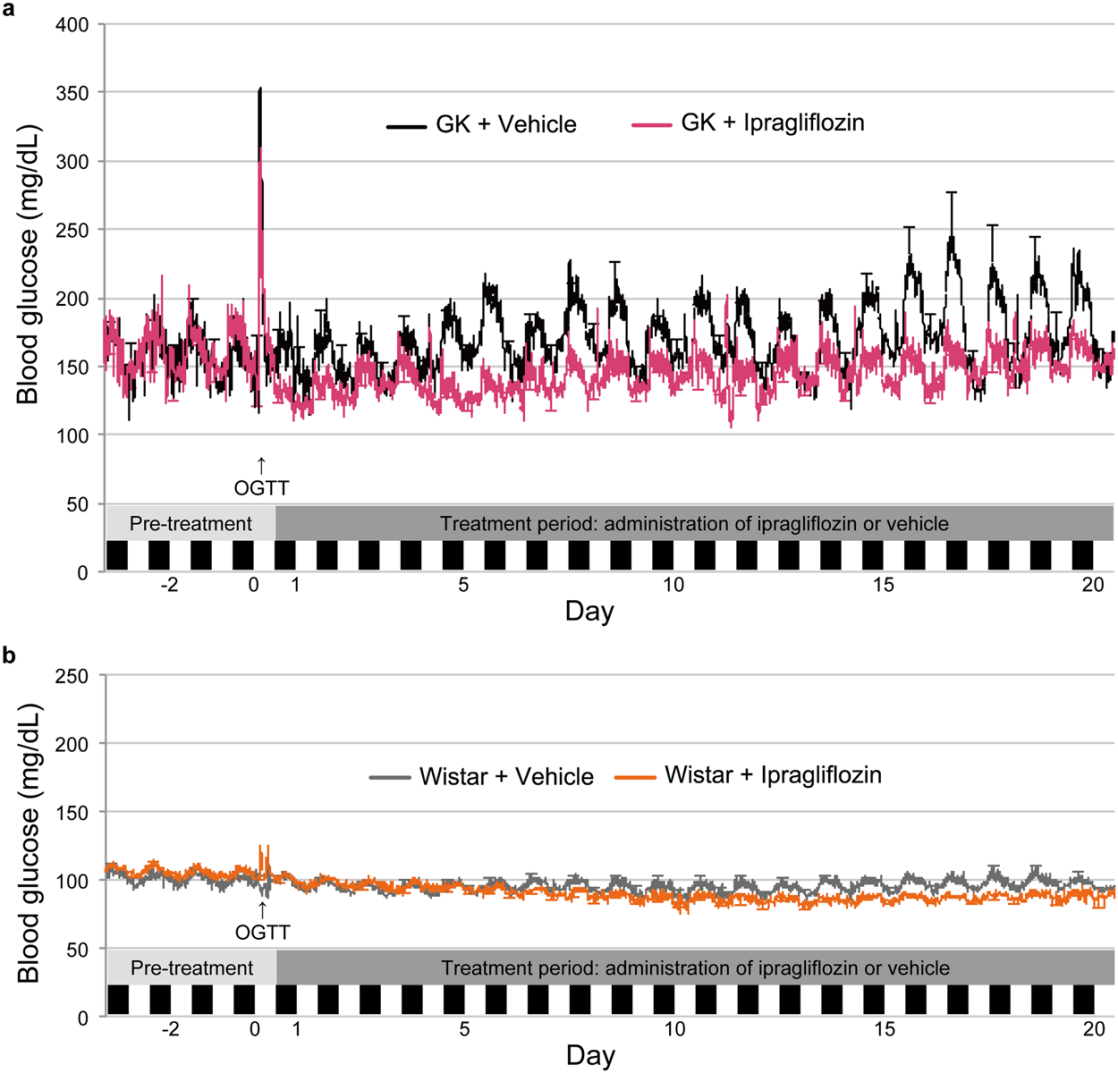
Changes in blood glucose as assessed by glucose telemetry during the experimental period treated with or without ipragliflozin. (**a**) Blood glucose (BG) of GK rats. (**b**) BG of Wistar rats. Error bars every 12 h indicate either the upper or lower s.e.m.

**Figure 3:**
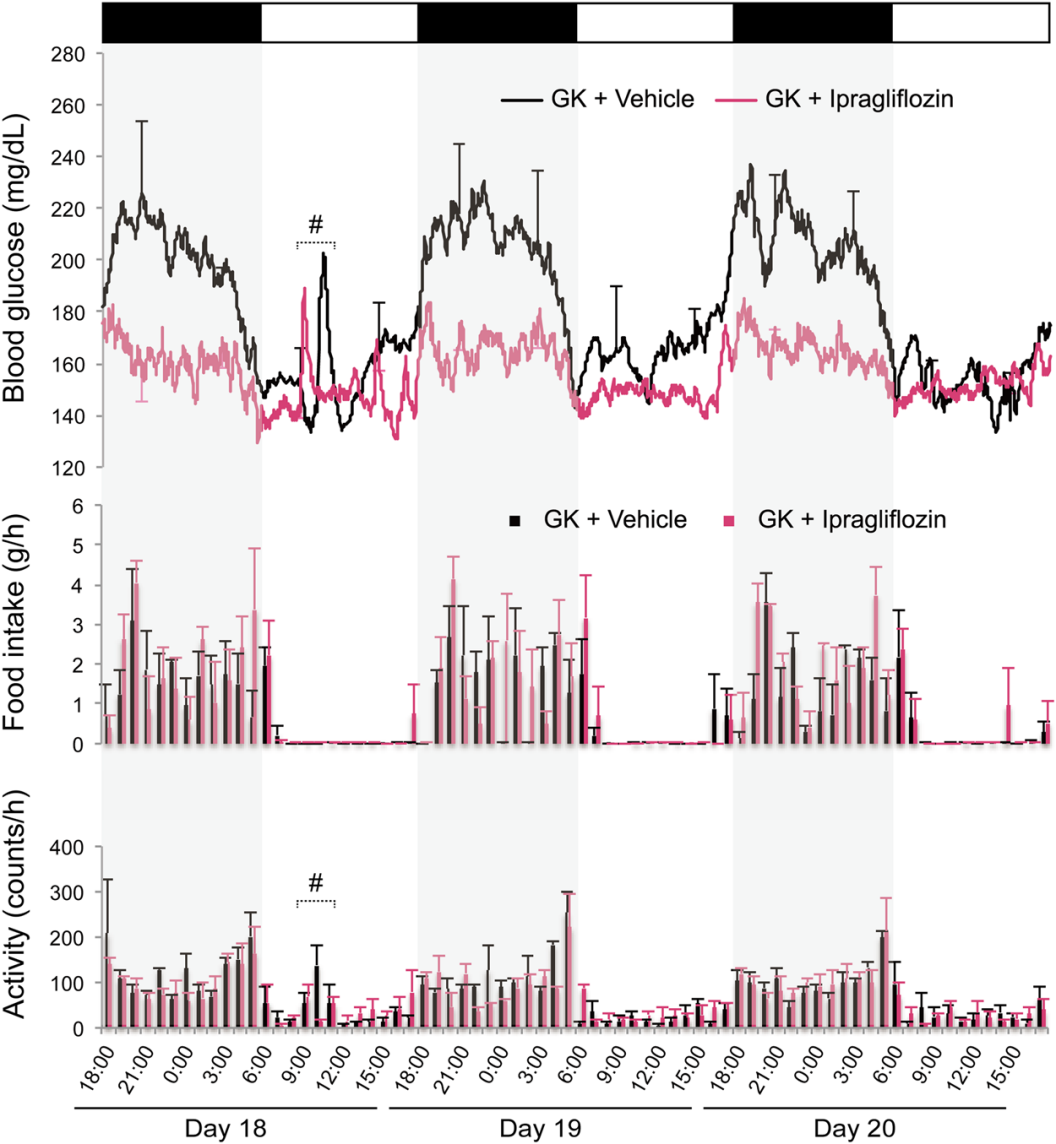
Simultaneous display of BG, food intake, and activity from days 18–20 in GK rats. Upper panel: BG as assessed by glucose telemetry. Error bars every 6 h indicate either the upper or lower s.e.m. Middle panel: Food intake measured every hour. Lower panel: Activity measured every hour. #Reactions to short-term handling stress, such as blood collection and measurement of body weight. Values are expressed as mean ± s.e.m. *n* = 3–4 per group.

**Figure 4:**
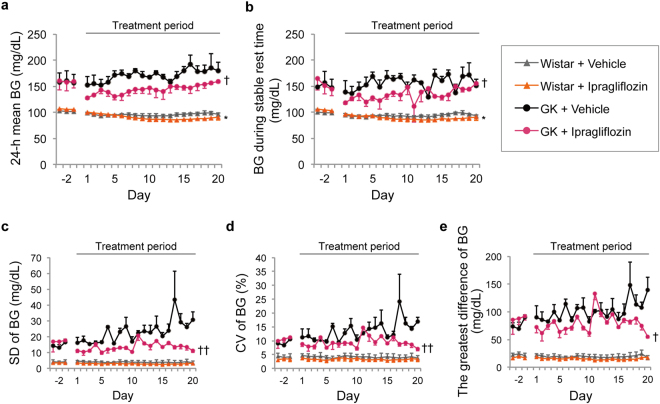
Effect of ipragliflozin on glycaemic profile as assessed by glucose telemetry in normal and GK rats. (**a**) 24-h mean BG. *P < 0.05 *vs*. Wistar + vehicle, days 15–18. ^†^ *P* < 0.05 *vs*. GK + vehicle, days 5, 6, 8, and 15. (**b**) BG during stable rest time. *P < 0.05 *vs*. Wistar + vehicle, day 17. ^†^ *P* < 0.05 *vs*. GK + vehicle, days 5, 14, and 15. (**c**) Standard deviation of BG. ^††^ *P* < 0.05 *vs*. GK + vehicle, days 3, 5, 6, 8, 15, 19, and 20. (**d**) Coefficient of variation of BG. ^††^ *P* < 0.05 *vs*. GK + vehicle, days 18–20. (**e**) Greatest difference of BG. ^††^ *P* < 0.05 *vs*. GK + vehicle, days 19 and 20. Values are expressed as mean ± s.e.m. *n* = 3–4 per group.

Blood glucose levels measured by continuous glucose telemetry during the entire period are shown in Fig. 2. Additionally, blood glucose, food intake, and activity from days 18–20 in GK rats are displayed in Fig. 3. Ipragliflozin significantly reduced the 24-h mean blood glucose level in normal and GK rats (days 15–18, *P* < 0.05; days 5, 6, 8, and 15, *P* < 0.05) (Fig. 4a). In the ipragliflozin-treated GK rats, 24-h mean blood glucose was reduced from baseline in the acute phase of ipragliflozin administration and then increased over time. Blood glucose during stable rest time slightly decreased in normal and GK rats (day 17, *P* < 0.05; days 5, 14, and 15, *P* < 0.05) (Fig. 4a). Measures of short-term glycaemic variability, such as the standard deviation (SD), coefficient of variation (CV), and greatest difference of blood glucose, were significantly lower in the ipragliflozin-treated GK rats than in the vehicle-treated GK rats (Fig. 4c–e). The difference in short-term glycaemic variability between the vehicle- and ipragliflozin-treated GK rats remained throughout the treatment period, despite the increase in 24-h mean blood glucose. In Wistar rats, ipragliflozin did not affect the short-term glycaemic variability.

### Ipragliflozin enhanced VO_2_ and energy expenditure in both normal and GK rats

**Figure 5:**
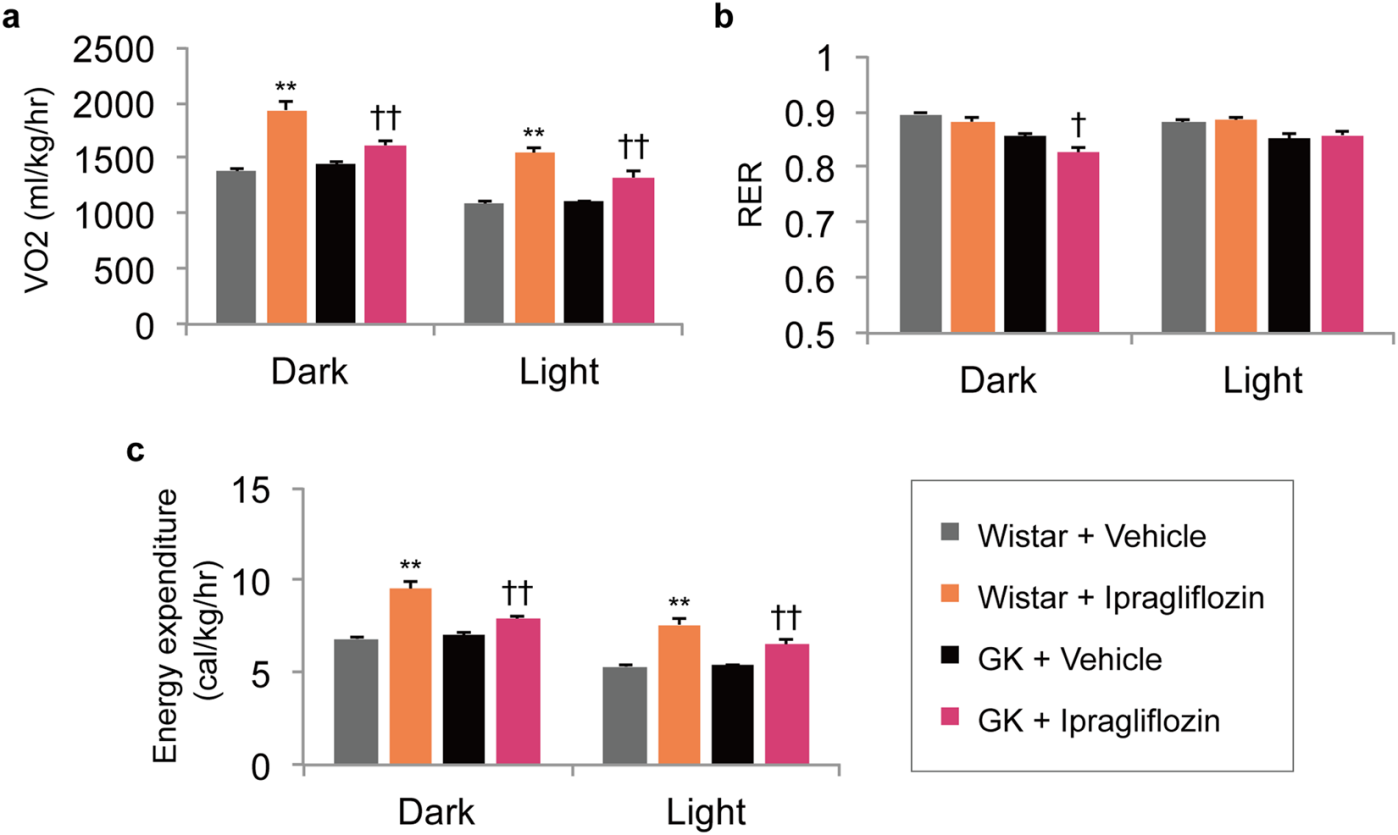
Effect of ipragliflozin on oxygen consumption, respiratory exchange ratio, and energy expenditure in normal and GK rats. (**a**) Oxygen consumption during dark and light periods. (**b**) Respiratory exchange ratio during dark and light periods. (**c**) Energy expenditure during dark and light periods. **P < 0.05 *vs*. Wistar + vehicle. ^†^ *P* < 0.05, ^††^ *P* < 0.01 *vs*. GK + vehicle. Values are expressed as mean ± s.e.m. *n* = 6 per group.

Ipragliflozin treatment for 20 days significantly increased VO_2_ and energy expenditure during the dark and light periods in both normal and GK rats, compared with those in vehicle-treated groups (Fig. 5a and c). Activity and body temperature also decreased in the ipragliflozin-treated normal rats. Meanwhile, the respiratory exchange ratio (RER) was reduced only during the dark period in the ipragliflozin-treated GK rats (Fig. 5b). This change is consistent with the changes in metabolic parameters, such as the circulating concentrations of triglyceride and ketone bodies.

### Ipragliflozin reduced body weight in normal rats during the entire treatment period and reduced the glycogen content in liver in GK rats

**Figure 6:**
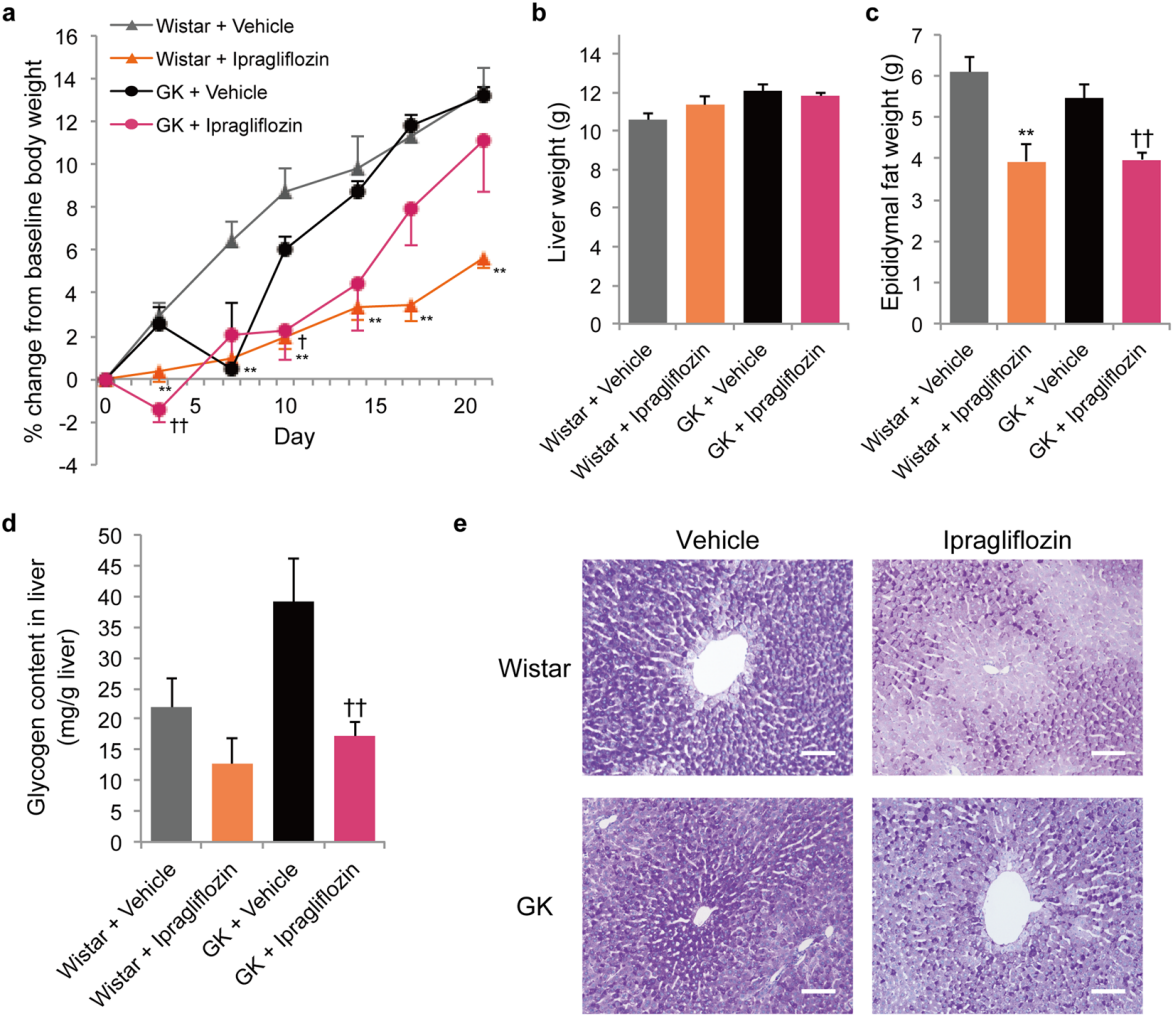
Effects of ipragliflozin on body weight, liver weight, epididymal fat weight, and glycogen content in liver in normal and GK rats. (**a**) Percentage change of body weight from baseline. (**b**) Liver weight. (**c**) Epididymal fat weight. (**d**) Glycogen content in liver. (**e**) Histological findings with periodic acid-Schiff staining of the liver. Scale bars, 100 μm. **P < 0.05 *vs*. Wistar + vehicle. ^†^ *P* < 0.05, ^††^ *P* < 0.01 *vs*. GK + vehicle. Values are expressed as mean ± s.e.m. *n* = 4–6 per group.

The body weight of the ipragliflozin-treated normal rats was suppressed during the entire treatment period compared with their body weight at baseline (Fig. 6a). However, in the ipragliflozin-treated GK rats, body weight was reduced only in the first half of the treatment period, compared with the vehicle-treated GK rats. In the latter half of the treatment period, the difference in the percentage change of body weight from baseline between the ipragliflozin-treated and vehicle-treated GK rats disappeared. In addition, the implantation of the telemetry device did not affect the gain of body weight (Supplementary Figure S2c).

The liver weight did not change in all groups (Fig. 6b). By contrast with the increase of body weight, the epididymal fat weight was reduced in both normal and GK rats (Fig. 6c).

Ipragliflozin treatment significantly decreased the glycogen content in liver in GK rats. In Wistar rats, ipragliflozin treatment tended to reduce the glycogen content in liver; however, there was no significant difference.

## Discussion

In the present study, ipragliflozin treatment induced urinary glucose excretion and increased food and water intakes, but decreased blood glucose within the normoglycaemic range in both normal and GK rats, resulting in enhanced basal energy expenditure. In addition, normoglycaemic rats underwent compensatory changes detected as altered circadian rhythms of food and water intakes, activity, and body temperature in the early phase of ipragliflozin treatment. Moreover, the present study is the first report of continuous blood glucose measurement for 4 weeks in rodents using glucose telemetry.

The dose of ipragliflozin used in this study induced the same amount of urinary glucose excretion in normal rats as in GK rats. Therefore, the urinary glucose excretion induced by SGLT2 inhibitors led to a negative energy balance in both normal and GK rats (Table 1). As compensatory mechanisms for lowered blood glucose, two representative mechanisms to produce blood glucose must be considered. First, liver glycogen, which is the main supply source of glucose in fasted state, was decreased by ipragliflozin treatment in this study (Fig. 6e). Similarly, SGLT2 inhibitors were reported to decrease glycogen content in liver^13,14^, suggesting that they promote glycogenolysis. The difference between normal and GK rats in their reactions to ipragliflozin might be attributed to differences in the amount of glycogen content or the times when tissue samples were collected^15,16^. Second, the upregulation of gluconeogenesis is essential, since the glycogen content in liver decreased regardless of the non-fasted state. In fact, SGLT2 inhibitors enhance endogenous glucose production, which is considered to be caused by an increased concentration of glucagon in plasma^17,18^.

Interestingly, in this study, energy expenditure was enhanced in both normal and GK rats. Under the condition of enhanced gluconeogenesis, basal energy expenditure is thought to increase, since gluconeogenesis requires adenosine triphosphate and involves a shift from anaerobic glycolysis to aerobic energy production, which requires an increase in oxygen consumption. However, inconsistent results have been reported regarding whether SGLT2 inhibitors enhance basal energy expenditure in rodent models^19–22^. Given the possibility that a high-fat diet decreases energy expenditure^23^, it is likely that different rodent models vary in their responses to SGLT2. In the literature, SGLT2 inhibitors have also been reported to attenuate the decrease in energy expenditure induced by a high-fat diet^20^. In any case, the energy deficit imposed by SGLT2 inhibitors-induced urinary glucose excretion is not like that caused by a low calorie intake^10^. Additionally, SGLT2 inhibitors promote lipolysis, reflecting a reduction in the RER. That effect could account for the decreases in triglyceride and epididymal fat weight that were observed in this study. However, the RER decreased during only the dark period in the ipragliflozin-treated GK rats, and the RER did not change during either the dark or light period in the ipragliflozin-treated normal rats (Fig. 5b), possibly suggesting that the utilisation of glucose is impaired during the active period, but not the inactive period, in non-obese GK rats.

Increased urinary glucose excretion and enhanced energy expenditure contribute to a negative energy balance associated with weight loss. By contrast, hyperphagia to maintain the level of blood glucose is one of the few countermeasures that can lead to a positive energy balance. In this study, ipragliflozin increased food intake in both normal and GK rats (Fig. 1a and b). However, it is unclear why food intake increased, nor is it clear why the increase of food intake in GK rats was higher than that in normal rats. At least two reports have demonstrated that ketone bodies act as a hunger signal to induce hyperphagia^24,25^. Since SGLT2 inhibitors promote the generation of ketone bodies^26^, SGLT2 inhibitor-induced ketone bodies might cause hyperphagia. Moreover, considering previous reports that streptozotocin-induced diabetic rats and GK rats exhibit hyperphagia^27–29^, the appetite of diabetic rats might react to various pharmaceutical stimuli.

Although blood glucose decreased from baseline despite some level of compensatory hyperphagia, the 24-h mean blood glucose in GK rats increased over time after ipragliflozin was administered (Fig. 4a). Nevertheless, ipragliflozin improved the short-term glycaemic variability in GK rats until the end of treatment period. Furthermore, ipragliflozin seems to have the effect of suppressing the “dawn phenomenon” (Figs 3 and 4b), which is an early morning increase in blood glucose in humans that is opposite to the day-night cycle in rodents^30^. Thus, there is a possibility that SGLT2 inhibitors may have a protective effect against organ damage induced by short-term glycaemic variability^31,32^. On the other hand, ipragliflozin did not affect the HbA1c levels of both Wistar and GK rats (Table 1). It has previously been reported that HbA1c of young GK rats is not high and is equivalent to that of Wistar rats^33^. Therefore, the assessment of blood glucose by glucose telemetry was useful at the ages of GK rats in this study. Additionally, these results suggest that calorie restriction plus SGLT2 inhibitors might be a valid clinical approach to achieve improved glycaemic control in the hyperglycaemic state^9^. However, it should be noted that treatment with calorie restriction plus SGLT2 inhibitors in the normoglycaemic state might lead to unsuitable conditions, such as evening hyperphagia and an excessive production of ketone bodies^34^.

Meanwhile, ipragliflozin greatly increased water intake in normal and GK rats (Fig. 1c and d). Because water intake behaviour, which is not measured by the locomotor activity of telemetry, seems to be a large action for rodents, it is likely that water intake behaviour increases energy expenditure, apart from the discussion about water-induced thermogenesis^35^.

Taken together, in GK rats treated with ipragliflozin for 20 days, hyperphagia could sufficiently compensate for the calorie loss caused by urinary glucose excretion and enhanced energy expenditure, resulting in a suppression of the reduction of body weight. However, ipragliflozin treatment for the longer period of 9 weeks decreased body weight in GK rats that were older than those used in this study, inducing a loss of fat mass^36^. By contrast, in normal rats treated with ipragliflozin for 20 days, urinary glucose excretion and enhanced basal energy expenditure exceeded the compensatory effect of hyperphagia, resulting in the reduction of body weight, which represented a net negative energy balance. The lower activity in ipragliflozin-treated normal rats was thought to occur in order to suppress further daily total energy loss, and subsequently led to a drop in activity-induced thermogenesis (Fig. 1e and f, Supplementary Figure S1c and d). Moreover, notably, the circadian rhythms of food and water intakes, activity, and body temperature shifted from the dark to light periods in the acute phase of ipragliflozin treatment (Supplementary Figure S1). These results could indicate that compensatory mechanisms such as gluconeogenesis and hyperphagia were partially activated within the first few days of treatment in the normoglycaemia state, and the response of these mechanisms became stronger during the treatment period. The reduction of energy expenditure in the acute phase of SGLT2 inhibitor treatment might reflect these conditions^14^.

This study has the following limitations: (1) To minimise stresses that could affect blood glucose, ipragliflozin was administrated by ad libitum feeding with an ipragliflozin-supplemented diet, rather than forced oral dosage. The content of ipragliflozin in the food was amended according to changes in the amount of daily food intake to maintain a constant daily dose; (2) Short-term handling stresses, such as body weight measurements and blood collections, were not excluded in the analysis of blood glucose. For that reason, these stresses might have affected the data for blood glucose, particularly short-term glycaemic variability; (3) The glucose telemetry devices used (HD-XG, Data Sciences International, New Brighton, MN, USA) could provide continuous measurements of blood glucose for only 4 weeks. To investigate the effect of ipragliflozin during a longer period, the development of improved glucose telemetry tools is needed. Despite these limitations, however, we believe that this study provides new and important insights into the effect of SGLT2 inhibitors.

In conclusion, ipragliflozin treatment resulted in increased food and water intakes to compensate for urinary glucose excretion, but led to decreased blood glucose as assessed by glucose telemetry in both normal and GK rats, resulting in an enhanced basal energy expenditure. In addition, ipragliflozin altered behavioural circadian rhythms in the acute phase of treatment in normoglycaemic rats.

## Methods

### Animals

All experimental procedures and sample collection methods were approved by the animal ethics committee of Jikei University (2016–043), and all experiments were carried out in accordance with the guidelines of the committee. Male Wistar and GK rats (280–350 g) were purchased from Sankyo Lab Service Corporation, Inc (Tokyo, Japan) at the age of 11 weeks and housed in a specialised polycarbonate cage, which automatically measures food and water intakes (FD-C700AS, MELQUEST, Toyama, Japan), with a wooden chip mat on the floor. Food and water were made available ad libitum for all rats. A standard diet (CE-2; 343 kcal/100 g, 12.6% energy as fat; CLEA Japan, Inc., Tokyo, Japan) was used in this study. The animal room was kept on a 12-h light/dark cycle (light 6:00 AM to 6:00 PM, dark 6:00 PM to 6:00 AM), at a constant temperature of 24 ± 2 °C and a relative humidity of 50 ± 10% throughout the experimental period.

### Experimental protocol

At the age of 12 weeks, all rats were assigned to two groups that received ipragliflozin (provided by Astellas Pharma Inc., Tokyo, Japan) mixed into their diet (0.008% [w/w]), or vehicle. The timeline of the experiment including telemetry analysis is shown in Supplementary Figure S3. Blood glucose was continuously measured by glucose telemetry, and food and water intakes were simultaneously measured every hour. Baseline body weight was set at day 0 and the percent change of body weight from baseline was measured twice a week. After 3 weeks of treatment, all rats were placed in individual metabolic cages on day 21 for the collection of 24-h urine samples. At the end of the experiment, blood samples were collected in the non-fasting state.

Indirect calorimetry, tissue collection, and histological analysis were performed in the other groups, independently of the groups with telemetry analysis, and the other groups were treated during the same period as the groups with telemetry analysis. On the final day of treatment after the measurement by indirect calorimetry, i.e., day 21, non-fasted rats were euthanised under isoflurane anaesthesia. The liver and epididymal fat were quickly removed from each animal and weighed. A part of the liver was frozen in liquid nitrogen to measure glycogen content, and the remaining part of the liver was fixed for histological analysis. Liver tissue sections were stained with periodic acid-Schiff.

### Glucose telemetry and assessment of glycaemic profile

At the age of 11 weeks, all rats underwent surgical implantation of a glucose telemetry device (HD-XG, Data Sciences International) into the abdominal aorta under intraperitoneal anaesthesia with a mixture of ketamine (50 mg/kg) and medetomidine (0.5 mg/kg). Blood glucose was measured by the tail vein twice per week and oral glucose tolerance tests were performed at the beginning and end of the treatments as a calibration for the glucose telemetry. The computer program (Dataquest^®^ ART Silver) provided with the telemetry device sampled calibrated values of blood glucose and body temperature as well as noncalibrated locomotor activity counts. Blood glucose was monitored by glucose telemetry and measured every 1 min for 4 weeks under the unrestricted and conscious condition. Body temperature was analysed as the mean every hour.

The blood glucose profile was analysed as the mean, SD, coefficient of variation (SD × 100/mean, %), and greatest difference (maximum value − minimum value) of blood glucose every 24 h. We defined “blood glucose during stable rest time” as the mean blood glucose during each hour from 15:00 PM, which was the period of each day with the lowest food intake (mean intake < 0.5 g/h) and activity (mean activity < 60 counts/h) during the treatment period.

### Biochemical assays

Blood glucose was measured using a glucometer (StatStrip^®^ Xpress; Nova Biomedical, Waltham, MA, USA). Blood biochemistry measurements and urinary glucose levels were performed at a laboratory of Oriental Yeast Co., Ltd. (Tokyo, Japan). Glycogen content in liver was measured by colorimetric assay using the Glycogen Assay Kit II (Abcam, Cambridge, UK).

### Indirect calorimetry

On day 20 of treatment with or without ipragliflozin, oxygen consumption was determined with an O_2_/CO_2_ metabolism measuring system (MK-5000RQ, Muromachikikai, Tokyo, Japan)^37^. Each ad libitum-fed rat was kept unrestrained in a sealed chamber with an air flow of 2.2 L/min for 20 h (light 6:00 AM to 2:00 PM, dark 6:00 PM to 6:00 AM). Air was sampled every 3 min, and the consumed oxygen concentration (VO_2_) and carbon dioxide production (VCO_2_) were calculated. The RER was calculated by dividing VCO_2_ by VO_2_. Metabolic calories (E) were calculated using the system software as follows: E (cal/kg/hr) = (1.07 × RER + 3.98) × VO_2_ × 60/body weight.

### Statistical analysis

Values were expressed as mean ± standard error of the mean. Two-group comparisons were performed using Student’s *t*-test. A *P* value of <0.05 was considered significant in all analyses. All statistical analyses were performed using SPSS Advanced Statistics version 22 software (IBM, Armonk, NY, USA).

## Electronic supplementary material

Supplementary Information
