# Supplementary material for: Time-dependent effects of ipragliflozin on behaviour and energy homeostasis in normal and type 2 diabetic rats: continuous glucose telemetry analysis
Source: Sci Rep. 2017 Sep 19;7:11906. doi: 10.1038/s41598-017-12106-y (PMC5605532; doi:10.1038/s41598-017-12106-y)

Supplementary Information

Time-dependent effects of ipragliflozin on behaviour and energy homeostasis in normal and type 2 diabetic rats: continuous glucose telemetry analysis

Hiroyuki Iuchi[1], Masaya Sakamoto[1], Daisuke Matsutani[1], Hirofumi Suzuki[1], Yosuke Kayama[2], Norihiko Takeda[3], Susumu Minamisawa[4] and Kazunori Utsunomiya[1]

[1] Division of Diabetes, Metabolism and Endocrinology, Department of Internal Medicine, The Jikei University School of Medicine, Tokyo, Japan

[2] Department of Cardiology, The Jikei University School of Medicine, Tokyo, Japan

[3] Department of Cardiovascular Medicine, Graduate School of Medicine, The University of Tokyo, Japan

[4] Department of Cell Physiology, The Jikei University School of Medicine, Tokyo, Japan

Address correspondence to:

Corresponding author: Masaya Sakamoto, MD, PhD

Division of Diabetes, Metabolism and Endocrinology, Department of Internal Medicine, Jikei University School of Medicine, Tokyo, Japan

Address: 3-25-8 Nishi-Shinbashi, Minato-ku, Tokyo 105-8461, Japan

E-mail: m-sakamoto@umin.ac.jp

Telephone number: +81-3-3433-1111

Fax number: +81-3-3578-9753

Figure legends

Supplementary Figure S1: Effects of ipragliflozin on (a) food intake, (b) water intake, (c) activity, and (d) body temperature during dark and light periods, displayed in Wistar (left panel) and GK (right panel) rats.

Supplementary Figure S2: Effects of telemetry on (a) food intake, (b) water intake, and (c) percentage change of body weight from baseline during dark and light periods in GK rats.

(a) Daily food intake during dark and light periods. # $P < 0.05$, ## $P < 0.05$ GK + vehicle *vs.* GK-Sham; dark period, days −2, −1, 3, and 12; light period, day −2. (b) Daily water intake during dark and light periods. # $P < 0.01$ GK + vehicle *vs.* GK-Sham; light period, day 7. (c) Percentage change of body weight from baseline. Values are expressed as mean ± s.e.m. $n = 4$ per group.

Supplementary Figure S3: Experimental timeline in the group with telemetry analysis.

Supplementary Figure S1: Effects of ipragliflozin on (a) food intake, (b) water intake, (c) activity, and (d) body temperature during dark and light periods, displayed in Wistar (left panel) and GK (right panel) rats.

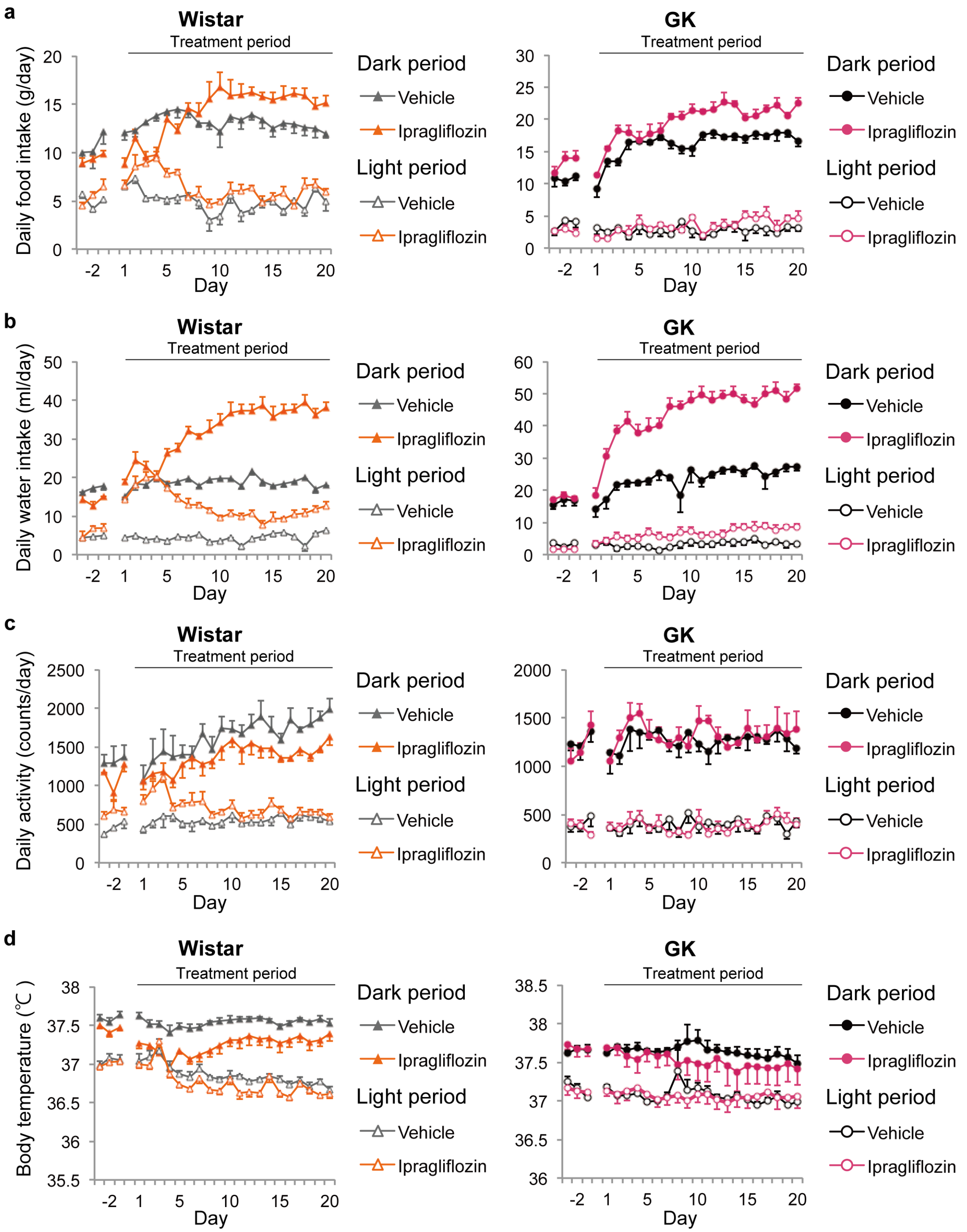

Supplementary Figure S2: Effects of telemetry on (a) food intake, (b) water intake, and (c) percentage change of body weight from baseline during dark and light periods in GK rats.

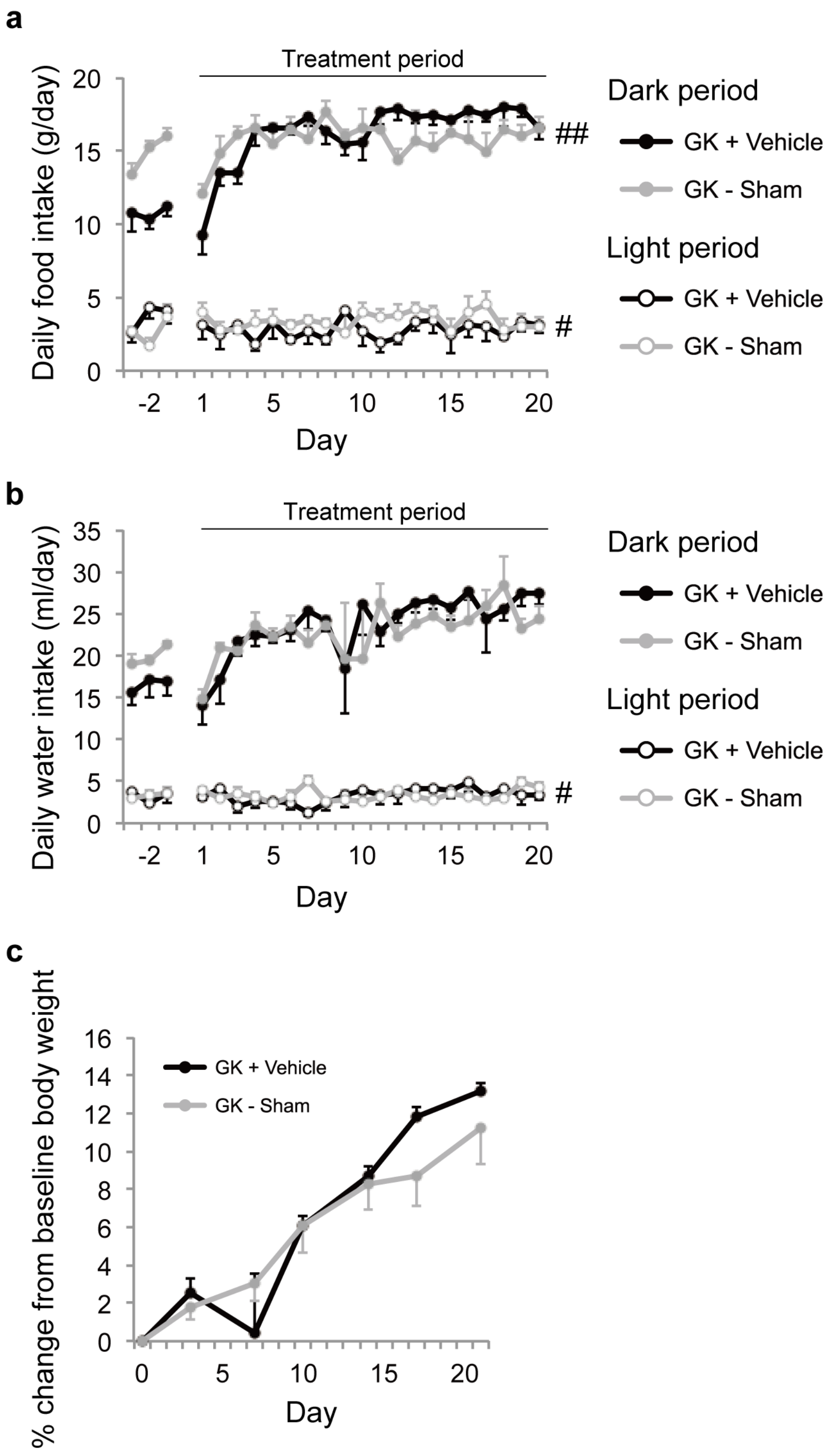

Supplementary Figure S3: Experimental timeline in the group with telemetry analysis.

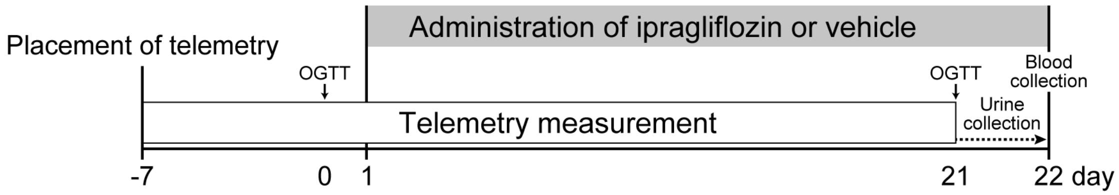

Supplement: Supplementary file 1 — Supplementary Information [file 41598_2017_12106_MOESM1_ESM.pdf]
